# Current challenges and proposed solutions to the effective implementation of the RTS, S/AS01 Malaria Vaccine Program in sub-Saharan Africa: A systematic review

**DOI:** 10.1371/journal.pone.0209744

**Published:** 2018-12-31

**Authors:** Christian Akem Dimala, Belmond Tse Kika, Benjamin Momo Kadia, Hannah Blencowe

**Affiliations:** 1 Acute Medicine Unit, University Hospitals of Leicester, Leicester, United Kingdom; 2 Health and Human Development (2HD) Research Network, Douala, Cameroon; 3 Department of Public Health, Universite Libre de Bruxelles, Brussels, Belgium; 4 Clinical Research Education, Networking and Consultancy Group (CRENC), Douala, Cameroon; 5 Faculty of Epidemiology and Population Health, London School of Hygiene and Tropical Medicine, London, United Kingdom; 6 Grace Community Health and Development Association, Kumba, Cameroon; 7 Department of Infectious Disease Epidemiology, London School of Hygiene and Tropical Medicine, London, United Kingdom; International Medical University, MALAYSIA

## Abstract

**Background:**

The Malaria Vaccine Implementation Program, coordinated by the World Health Organization, intended to initiate the roll-out of the RTS, S/AS01 malaria vaccine in 3 sub-Saharan African countries in 2018. With sub-optimal implementation, the effectiveness of this vaccine in routine clinical use could be significantly lower than its measured efficacy in randomized trials. This study had as objectives to systematically review and summarize published studies addressing the challenges faced during the implementation phase of malaria vaccination programs and randomized trials conducted in sub-Saharan Africa. The review also sought to report proposed solutions to the challenges identified.

**Method:**

This was a systematic review of studies published between 1947 and 2017. Medline, Embase and the Cochrane library databases were searched. Of the 365 studies retrieved, 8 eligible studies reported on challenges of implementing a malaria vaccine in sub-Saharan Africa and possible solutions to these challenges. Data were abstracted from the eligible studies and a qualitative synthesis was done.

**Results:**

The 8 studies included in the review had a total of 6189 participants and used a variety of methodologies (3 qualitative, 1 quantitative, 3 mixed method studies and 1 clinical trial review). There was an overall positive acceptance towards the new malaria vaccine (n = 6/8 studies), with a mean acceptance rate of 86.1% (95% CI: 62.0–110.2, n = 2). The main challenges to vaccine receptivity were: inadequate community engagement due to lack of information about the vaccine (n = 6), fear of the vaccine’s side effects (n = 5), inefficient delivery of vaccination services to children (n = 4), and sub-optimal quality of the health services (n = 3). Main themes identified from the proposed solutions consisted of the following: using dynamic communication models and trusted sources for delivering vaccine-related health information to the communities (n = 6), community engagement at both national and district level (n = 6), implementing the new vaccine services alongside the existing health services already delivered (n = 6).

**Conclusion/Recommendations:**

Effective implementation of the malaria vaccine program requires careful consideration of the socio-cultural context of each community. The RTS, S/AS01 malaria vaccine acceptance and uptake may be significantly enhanced if caregivers’ perceptions about vaccines and their importance are adequately fine-tuned. In order to achieve these, community participation and the provision of adequate information in an acceptable form via reliable communication channels seem to be imperative.

## Introduction

Significant progress has been made worldwide in controlling malaria over the past decades, with a reported 62% reduction in malaria-related mortality between 2000 and 2015 [1]. Despite these achievements, malaria remains a major public health concern. In 2015, it was estimated that there were 212 million cases of malaria and 429,000 malaria-related deaths worldwide, with sub-Saharan Africa (SSA) bearing the greatest burden [1]. Current malaria prevention measures in SSA include chemoprevention in pregnant women and infants, vector control using insecticide treated bed nets and indoor residual spraying. Despite these measures, malaria remains a major cause of mortality in this part of the world especially among children under five years of age.

Several malaria vaccine projects have been underway in a bid to address the huge burden posed by malaria. In 2015, the European Medicines Agency for the immunization of children against malaria approved the RTS, S/AS01 vaccine [2]. This is currently the most clinically advanced malaria vaccine [2]. Phase 3 clinical trials conducted in various sites in Africa showed that the RTS, S/AS01 vaccine has a protective efficacy of 45% in children in the first twenty months after vaccination [3,4]. In 2018, the World Health Organization through a large scale pilot malaria vaccine implementation program (MVIP) aimed to introduce this vaccine in three sub-Saharan countries (Ghana, Kenya, Malawi) [5]. However, the effectiveness of this vaccine in routine clinical use might be much lower than in randomized trials because of the effect of external factors such as vaccine receptivity, storage and administration. Optimal implementation of the vaccine program is therefore required to ensure maximum efficacy. To achieve this, it is important to investigate potential challenges during the implementation phase of the vaccination programs and take the appropriate measures to address these challenges. This study aimed to review existing evidence on the challenges to the implementation of earlier malaria vaccination programs in SSA and to explore proposed solutions to these challenges.

## Methods

### Search strategy and selection criteria

This was a systematic review of studies on the implementation of malaria vaccine programs and randomized trials in SSA. The studies reviewed were published between January 1947 and December 2017. Databases searched included: Cochrane library, Embase and Medline. An extensive search strategy was developed by combining the subject heading search and the keywords free-text search (Table 1). Articles retrieved from the search were saved on the Zotero referencing manager (version 4.0.29.22). The reference lists of appropriate studies were also reviewed to identify additional studies. The titles and abstracts of all identified studies were then screened by 2 independent investigators (CAD and BTK). Following the titles and abstracts screening, the full texts of retained studies were independently reviewed by both investigators to identify eligible studies.

**Table 1:** Search strategy.

Search #	Search words
**1**	RTS, S [Mesh]
**2**	RTS vaccine OR RTS,S vaccination OR RTS,S implementation OR RTS,S vaccination program* OR RTS,S immunization OR RTS,S immunization OR RTS,S immunisation program* OR RTS,S immunization program* OR Malaria vaccine OR Malaria vaccination OR Malaria implementation OR Malaria vaccination program* OR Malaria immunization OR Malaria immunization OR Malaria immunisation program* OR Malaria immunization program*
**3**	#1 OR #2
**4**	Challenges OR difficulties OR shortcomings OR problems OR complication OR drawback OR hindrance OR impediment OR barrier OR disadvantage OR obstacle
**5**	Sub-Saharan Africa [MeSH]
**6**	Africa OR Algeria OR Angola OR Benin OR Botswana OR Burkina Faso OR Burundi OR Cameroon OR Canary Islands OR Cape Verde OR Central African Republic OR Chad OR Comoros OR Congo OR Democratic Republic of Congo OR Djibouti OR Egypt OR Equatorial Guinea OR Eritrea OR Ethiopia OR Gabon OR Gambia OR Ghana OR Guinea OR Guinea Bissau OR Ivory Coast OR Cote dIvoire OR Jamahiriya OR Jamahiryia OR Kenya OR Lesotho OR Liberia OR Libya OR Libia OR Madagascar OR Malawi OR Mali OR Mauritania OR Mauritius OR Mayotte OR Morocco OR Mozambique OR Mocambique OR Namibia OR Niger OR Nigeria OR Principe OR Reunion OR Rwanda OR Sao Tome OR Senegal OR Seychelles OR Sierra Leone OR Somalia OR South Africa OR St Helena OR Sudan OR Swaziland OR Tanzania OR Togo OR Tunisia OR Uganda OR Western Sahara OR Zaire OR Zambia OR Zimbabwe OR Central Africa OR Central African OR West Africa OR West African OR Western Africa OR Western African OR East Africa OR East African OR Eastern Africa OR Eastern African OR North Africa OR North African OR Northern Africa OR Northern African OR South African OR Southern Africa OR Southern African OR subSaharan Africa OR subSaharan African OR sub-Saharan Africa OR sub-Saharan African
**7**	#5 OR #6
**8**	#3 AND #4 AND #7

The studies included were:

- Those published in English between January 1947 (earliest date of databases) and June 2017.
- Quantitative and qualitative studies and reviews with data on the challenges to the implementation of malaria vaccination interventions in SSA and the proposed solutions.
- Interventional studies, observational studies and reviews.

Studies excluded were:

- Those with insufficient data on the challenges or solutions to the implementation of malaria vaccine projects in SSA.
- Conference abstracts, reports and bulletins.

### Data analysis and synthesis

Data was extracted from eligible studies by the principal investigator (CAD). The extracted data was saved on a Microsoft Excel 2016 form and subsequently double-checked for accuracy by a second investigator (BTK). Data extracted consisted of: First author name, publication year, journal reference, country and place of the study, year of study, study setting and design, sample size, study population, vaccine acceptance rates, study limitations, reported challenges to program implementation and proposed solutions to the challenges. The two principal outcomes of interest were: challenges encountered, and proposed solutions. A thematic content analysis approach was adopted for data analysis and synthesis. CAD and BTK developed the initial coding framework on Microsoft Excel 2016 by reading through the eligible studies and identifying main themes from these studies. The main themes were developed based on the two main outcomes of interest: challenges to the implementation of malaria vaccination programs and proposed solutions. The coding framework was then progressively amended to incorporate more main themes and sub-themes that emerged as each eligible study was reviewed. The overall vaccine acceptance rate was calculated as the mean of the acceptance rates reported by the individual studies. The quality of qualitative studies was assessed using the critical appraisal skills program (CASP) checklist [6], while that of the interventional and observational studies was assessed using their respective quality assessment tools as per the National Health Institute (National Heart, Lung, and Blood Institute) [7]. Overall study quality was rated as either good, fair or poor. A narrative approach was used to summarize the abstracted data. This review was reported in accordance with the Preferred Reporting Items for Systematic Reviews and Meta-Analysis (PRISMA) 2009 guidelines (See S1 Checklist for more details). A protocol of this review is available in PROSPERO (CRD42017077479).

## Results

### Overview of search results and quality of studies

**Fig 1:**
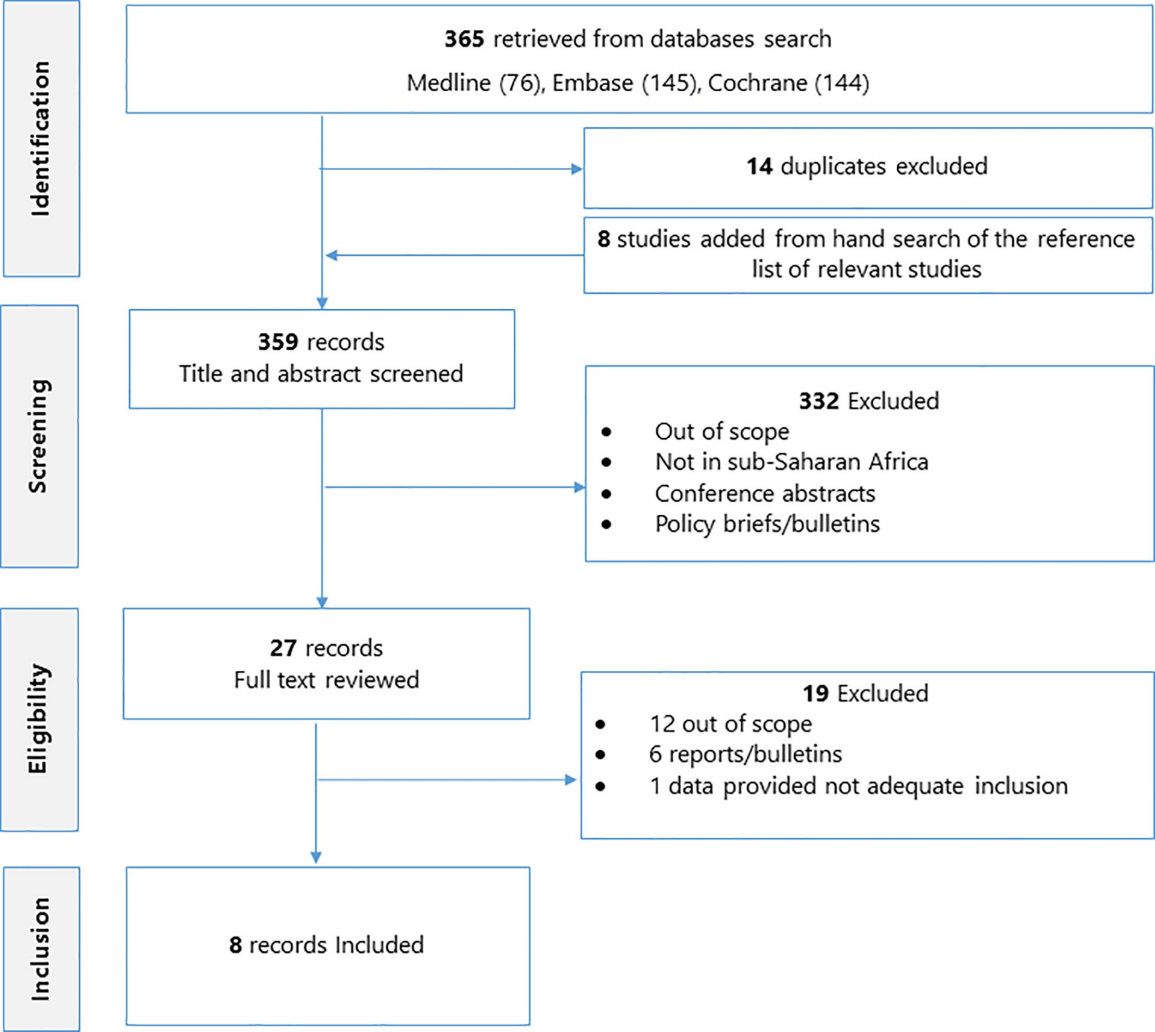
PRISMA flow chart illustrating study identification and selection.

The search strategy returned a total of 365 studies (Fig 1). Twenty-seven studies were retained for full text review after removal of duplicates, review of the reference list of relevant studies to identify eligible studies, and screening of study titles and abstracts. Eight studies were eligible for inclusion in the synthesis and had a total of 6189 participants. The main study characteristics are summarized on Table 2 (See S1 Table for more details). Three of these 8 studies had an exclusively qualitative design involving in-depth interviews, focus group discussions, semi-structured observations and exit interviews [8–10]. One study was exclusively observational, involving exit interviews [11]. Three of the eight studies used mixed methods: one combined a randomized trial with a qualitative design [12] and two combined cross-sectional and qualitative designs [13,14]. One of the eight studies was a clinical trial review [15]. Half of the studies were carried out in East Africa, three were from West Africa and one was from Southern Africa. Five of the studies were from both rural and urban settings [9–12,14] and two were exclusively from rural settings [8,13]. Of the 6189 participants, 1157 were involved in the qualitative studies. These included parents, caregivers, health professionals, community leaders, teachers, religious leaders, and other key stakeholders.

**Table 2:** Study characteristics.

First author (publication year)	Country	Study year	Study Design	Participants
Afolabi (2014) **[15]**	Gambia	2013	Clinical trial review	136
Angwenyi (2014) **[12]**	Kenya	2009–2011	RCT/Qualitative	561
Bingham (2012) **[8]**	Mozambique	2010	Qualitative	200
Febir (2013) **[13]**	Ghana	2007	Cross-sectional/Qualitative	625
Meñaca (2014) **[9]**	Ghana	2012–2013	Qualitative	286
Mtenga (2016) **[14]**	Tanzania	2013	Cross-sectional/Qualitative	2144
Ojakaa (2011) **[10]**	Kenya	2009	Qualitative	234
Ojakaa (2014) **[11]**	Kenya	2010	Cross-sectional	2003

RCT-Randomised controlled trial

Among the studies with a qualitative component, four were of good quality [8,9,12,14] and two were of fair quality [10,13] (S2 Table). The single randomized trial was of good quality [12] (S3 Table). Regarding studies with observational designs, two were of good quality [13,14] and one was of fair quality [11] (S4 Table).

### Malaria vaccine acceptance

Six studies reported an overall positive acceptance to the introduction of the malaria vaccine from the study participants [8–11,13,14]. Only two of these six studies reported numeric vaccine acceptance rates of 84.2% [14] and 88% [11] respectively, giving a mean vaccine acceptance rate of 86.1% (95% CI: 62.0–110.2, n = 2).

### Challenges to vaccine receptivity

Up to 75% of the studies (n = 6/8 studies) reported inadequate community engagement resulting from the lack of information about the vaccine [8–12,14]. With regards to knowledge about the malaria vaccine, a study found that up to 90% of its participants were aware that malaria can be prevented through vaccination [13], while another study reported that 90% of participants in its quantitative component were aware that vaccination is beneficial [14].

Fear of the vaccine’s side effects was reported as a challenge by 62.5% of the studies (n = 5) [8–11,14].

Half of the studies (n = 4) reported the inefficient delivery of the child immunization services as a major barrier to adequate vaccine implementation [8–10,15]. Inefficient delivery was linked to significant distances to vaccination sites [8,10,15], long queues and long hours of service at the vaccination sites [8,10].

An overall low quality of the services provided in the health facility was reported in 37.5% of the studies (n = 3) [8,10,11]. This low quality was linked to the lack of health supplies at hospitals, unresponsive hospital staff, and the poor communication between hospital staff and patients.

The vaccine acceptance was also found to be significantly affected by the health decision-maker in the household and the decision maker’s relationship with the child to be vaccinated [8,10,11].

Other important challenges reported by single studies were: the reduction in attendance of clinics after nine months [9], the partiality of the protection provided by the vaccine [8], the difficulties with storage of the vaccines [15], as well as socio-cultural practices and religious denominations against vaccination [10]. Table 3 summarizes the challenges to vaccine receptivity identified from the eligible studies.

**Table 3:** Identified themes regarding challenges to vaccine receptivity.

Challenge	Studies	Authors (publication year)
**Inadequate community engagement**		
Lack of information on vaccine	7	Bingham (2012) [8], Meñaca (2014) [9], Mtenga (2016) [14], Ojakaa (2011) [10], Ojakaa (2014) [11], Angwenyi (2014) [12], Febir (2013) [13]
**Fear of vaccine side effects**		
General fear that vaccines or injections might harm the child	5	Bingham (2012) [8], Meñaca (2014) [9], Mtenga (2016) [14], Ojakaa (2011) [10], Ojakaa (2014) [11]
**Inefficient delivery of the child immunization services**		
Significant distance to vaccination services, the long queues, and the hours of services	4	Bingham (2012) [8], Meñaca (2014) [9], Ojakaa (2011) [10], Afolabi (2014) [15]
**Quality of overall health service at health facility**		
Lack of health supplies, unresponsive hospital staff, poor communication between hospital staff and patients	3	Bingham (2012) [8], Ojakaa (2011) [10], Ojakaa (2014) [11]
**Health decision-making in households**		
Relationship of the caregiver to the children	3	Bingham (2012) [8], Ojakaa (2011) [10], Ojakaa (2014) [11]
**Other reasons**		
Reduced clinic attendance after 9 months Partiality of the vaccine protection Difficulties with vaccine storage Traditional cultural practices	4	Bingham (2012) [8], Meñaca (2014) [9], Afolabi (2014) [15], Ojakaa (2011) [10]

### Proposed solutions to the challenges reported

Solutions proposed to the problem of community engagement and lack of information on the vaccine included the use of trusted sources for delivering health information, involving stakeholders in planning and vaccine implementation at both the national and district levels, translating information about the vaccine into local languages, and involving local leadership in the selection and design of communication messages [8,9]. Other potential solutions highlighted in two studies were to address socio-cultural aspects (religion, ethnicity, occupation and region) that influence the decision of parents and caregivers and to use proper communication strategies to clarify the expectations of stakeholders prior to the introduction of the malaria vaccine [10,14]. Other strategies proposed were to target specific segments of child caregivers (residents of regions with low acceptance, service providers in health facilities, older caregivers, less educated) with relevant messages [11], and to embed community engagement activities in already existing structures and activities [12]. Implementing the malaria vaccine alongside the package of services of already well-established programs was proposed as a solution to increase vaccine uptake [9]. With regards to concerns about the vaccine’s side effects, effective communication on health information about the vaccine was suggested [8,9].

Providing vaccines free of charge at the clinics alongside other incentives such as bed nets was suggested as a solution to increase vaccine uptake despite the suboptimal quality of care delivered at health facilities [10]. Solutions to the challenges reported are summarized on Table 4. Other generic solutions proposed to the individual challenges mentioned were the development of cold-chain free vaccines for resource-constrained settings and the development of a sustainable “in-house” dry ice production for vaccine storage during transportation to the vaccination services [15].

**Table 4:** Proposed solutions identified from eligible studies.

Solutions	Authors (publication year)
**Inadequate community engagement**	
1. Using trusted sources for delivering health information, 2. Involving stakeholders in planning and implementation at all levels 3. Translating information into local languages 4. Involving local leadership in the design of communication messages 5. Using communication strategies to clarify questions and expectations of stakeholders prior to or parallel with the introduction of the vaccine 6. Targeting specific segments of child caregivers with relevant messages	Bingham (2012) [8], Meñaca (2014) [9], Mtenga (2016) [14], Ojakaa (2011) [10], Ojakaa (2014) [11], Angwenyi (2014) [12], Febir (2013) [13]
**Fear of vaccine side effects**	
1. Effective communication on health information about the vaccine using dynamic communication models that engage caregivers of children	Bingham (2012) [8], Meñaca (2014) [9],
**Inefficient delivery of the child immunization services**	
1. Implementing the malaria vaccine alongside the package of services already offered at welfare clinics	Meñaca (2014) [9]
**Quality of overall health service at health facility**	
1. Providing vaccines free of charge at the clinics and alongside other incentives such as bed nets	Ojakaa (2011) [10]
**Other reasons**	
1. Development of cold-chain free vaccines 2. Development of “in-house” dry ice production for vaccine storage	Afolabi (2014) [15]

## Discussion

This study is the first to assess a broad array of potential challenges to the implementation of malaria vaccine programs in SSA. The study also examines a wide range of solutions to the observed challenges. Moreover, the findings of this study are relevant to the MVIP, tasked with implementing the RTS, S/AS01 vaccine in 3 sub-Saharan African countries. These findings were obtained via a systematic review of published studies. In all, 8 studies (with a total of 6189 participants) were retained for the review. We noted an overall positive acceptance of a malaria vaccine. Inadequate community engagement, lack of information on the vaccine, concerns regarding the vaccine’s side effects, and inefficient delivery of the vaccination services were identified as the major challenges to vaccine acceptance. Improved community participation and delivery of health information through trusted communication channels were proposed as potential solutions.

According to the health belief model, individuals’ perceptions of diseases and beneficial effects of interventions that serve to counter diseases are key determinants of people’s responses to health interventions [16]. Previous studies conducted in diverse sub-Saharan African settings found that malaria was generally perceived as a major public health problem [17]. Therefore, it could be inferred that vaccination against malaria is perceived as an important preventive measure by the sub-Saharan African population and this could explain the overall high acceptance rate noted in our review.

Inadequate community engagement emerged as the most reported challenge. In recent years, the role of community engagement as a fundamental component in the implementation of contemporary clinical research and community-based interventions has been increasingly recognized [18–21]. However, the effective application of community engagement is significantly constrained by several factors such as identifying legitimate stakeholders of interest and determining their extent of involvement [12]. Making use of well-established programs with a satisfactory level of community engagement can enhance the acceptance of new interventions such as the malaria vaccine [9]. The already existing expanded program of immunization, for example, was found to be effective in increasing the community response to the newly introduced intermittent preventive treatment of malaria for infants in Mozambique [22]. Using recognized government-sponsored health information channels and community leaders as reliable information relay points has been proposed as a means to improve community engagement [8]. Also, minimizing barriers to understanding by translating and communicating available information in local languages have been reported as being effective in improving community engagement and vaccine acceptance [13].

Fears associated with vaccine side effects remain a significant barrier to the acceptance of the malaria vaccine [8–11,14]. Vaccination has previously been associated with the transmission of other diseases, as was observed in northern Nigeria with the polio vaccine [23]. Based on the health belief model, the benefits of the malaria vaccine could be poorly perceived because of concerns over its side effects. This could have a negative impact on the effectiveness of the malaria vaccination program. Information relayed to communities should therefore emphasize on the safety of the malaria vaccine. The implementation of post-vaccination support services to deal with all cases of adverse effects should also be considered.

Two closely related determinants of vaccine acceptance consistently reported in these studies were the inefficiency in the delivery of the child vaccination services and the overall quality of the health services offered at health facilities [8–10,15]. Care-givers are more likely to take their children to health facilities for them to be vaccinated if they are satisfied with the quality of the services offered at these health facilities [10,11]. Thus, improvements in the quality of services offered at health facilities including vaccination services appear to be fundamental to boosting vaccine acceptance.

A limited number of people have access to quality healthcare services in SSA due to the high proportion of people living below the poverty threshold. Making vaccination free of charge, and even using incentives to boost attendance of vaccination sessions have been observed in some instances [10]. However, the sustainability of such methods remains questionable. Nevertheless, cost-independent factors that negatively affect hospital attendance and access to vaccination are also likely to be encountered. An example of this, as reported by Meñaca *et al* in a study conducted in Ghana, is the observed reduction in hospital attendance of women and their children after the age of 9 months due to their perceived completion of the vaccination calendar [9]. The possibility of a similar trend in other sub-Saharan African countries should be considered and addressed.

The partiality of the protection conferred by the RTS, S/AS01 malaria vaccine and the waning of this protective efficacy over months after vaccination were reported as potential barriers to vaccine acceptance in a single study [11]. In contrast, these were not found to negatively affect the vaccine acceptance in 4 studies in which the communities perceived the vaccine as a complement rather than a substitute to the other malaria prevention measures [8–10,14]. Ensuring communities have the right perception about the role of vaccines is therefore important for adequate vaccine acceptance.

Religious and socio-cultural factors were noted to affect the receptivity of the vaccine to different extents across the studies. Febir *et al* in West Africa (Ghana) identified no religious beliefs or socio-cultural practices that negatively affected the acceptance of the malaria vaccine and went on to suggest the possibility of using religious leaders to easily introduce the malaria vaccine [13]. On the other hand Mtenga *et al* in East Africa (Tanzania) reported a variable acceptance of vaccination across various religious denominations, with a greater receptivity among Christians compared to Muslims [14].

It is worth noting that despite all these mentioned barriers to the uptake of the malaria vaccine, all six studies that reported on vaccine acceptance found an overall positive acceptance of the vaccine among participants. The interpretation of the findings of this review should take into consideration some limitations. Only studies published in English were included in the review and this could have potentially led to some selection bias. Also, the endemicity of malaria, and the socio-cultural and economic contexts vary widely across the continent. The findings of this study might therefore not be generalizable to the entire continent. Nevertheless, the results of this study highlight important considerations for the planned RTS, S/AS01 vaccine implementation in the selected 3 countries: Ghana, Kenya, and Malawi. Five of the studies included in this review are from two of these countries (Ghana and Kenya), and two of the studies are from countries (Tanzania and Mozambique) sharing borders with Malawi. This potentially approximates the findings of this review to the realities expected to be encountered on the field during the implementation of the malaria vaccine in these countries.

## Conclusion

The precise challenges in implementing a malaria vaccine in randomized trial conditions vary from one community to another and are determined by several factors including the socio-cultural context specific to that community. However, some common factors were seen across the 8 populations from the five countries included in this review. Overall, vaccine acceptance and uptake can be significantly enhanced if caregivers’ perceptions about vaccines and their importance is adequately fine-tuned. This can be achieved through adequate community participation and by providing information in an acceptable form via reliable communication channels.

## Supporting information

S1 Checklist PRISMA checklist for study on current challenges and proposed solutions to the effective implementation of the RTS, S/AS01 malaria vaccine program in sub-Saharan Africa: A systematic review. (PDF)

S1 Table Summary of included studies. (PDF)

S2 Table Qualitative study quality assessment using the critical appraisal skills program (CASP) checklist. (PDF)

S3 Table Quality assessment tool for the randomized trial. (PDF)

S4 Table Quality assessment tool for the cross-sectional studies. (PDF)

## Abbreviations

MVIP: Malaria Vaccine Implementation Program
SSA: Sub-Saharan Africa
