# Supplementary material for: Current challenges and proposed solutions to the effective implementation of the RTS, S/AS01 Malaria Vaccine Program in sub-Saharan Africa: A systematic review
Source: PLoS One. 2018 Dec 31;13(12):e0209744. doi: 10.1371/journal.pone.0209744 (PMC6312235; doi:10.1371/journal.pone.0209744)
Supplement: S1 Table — (PDF) [file pone.0209744.s002.pdf]

S1 Table. Summary of Included studies

Author	Place in country	Setting	Design (Sample size)	Study population	Challenges reported	Proposed solution	Limitations	Study quality
Afolabi MO (2014)		Rural	Clinical trial review (136)	Infants	Significant distance to vaccination services<br>Difficulties with storage of vaccines (cold chain)	Development of cold-chain free vaccines for resource-constrained settings<br>Development of "in-house" dry ice production for vaccine storage during transportation to the vaccination services	-	-
Angwenyi V (2014)	Kilifi	Urban and Rural	Qualitative (257)<br>RCT (904)	Parents, field workers, health facility staff, community leaders for the qualitative part.<br>Children: 5 - 17 months and 6 - 12 weeks old for the RCT	Inadequate community engagement/<br>Lack of information about the vaccine	embedding community engagement activities in existing structures and activities	-	Good
Bingham A (2012)	Chókwè and Massinga Districts	Rural	Qualitative (200)	Parents, caregivers, health providers, religious leaders, traditional healers, traditional birth attendants, and leaders of NGOs	Inadequate community engagement<br>Fear of vaccine side effects<br>Significant distance to vaccination services, long queues<br>Poor quality of services at health facilities	Trusted sources for delivering health information<br>Involving stakeholders in planning and implementation at all levels<br>Translation of information into local languages<br>Involving local leadership in the design of communication messages	Criterion-based sampling techniques to meet objectives, generalizability of results, social desirability bias.	Good
Febir LG (2013)	Kintampo North and South districts	Rural	Qualitative (159)<br>Cross-sectional (466)	Community members, health professionals and key stakeholders	-	Patients already had good knowledge about vaccine	Interviews not conducted in English but local language<br>Generalizability of results	Fair

Continued…

S1 Table. Summary of Included studies (Continued…)

Author	Place in country	Setting	Design (Sample size)	Study population	Challenges reported	Proposed solution	Limitations	Study quality
Meñaca A (2014)	Ashanti and Upper East districts	Urban and Rural	Qualitative (286)	Parents, relevant community members, health administrators, health professionals, formal and informal leaders	Lack of information about the vaccine	Trusted sources for delivering health information<br>Involving stakeholders in planning and implementation at all levels<br>Translation of information into local languages<br>Involving local leadership in the design of communication messages	-	Good
Mtenga S (2016)	12 Districts of Tanzanian mainland		Qualitative (21)<br>Cross-sectional (2123)	Primary school teachers, religious leaders, community health workers, health care professionals, scientists, mothers of children	Inadequate community engagement/<br>Lack of information about the vaccine	Communication strategy to clarify the questions and expectations of stakeholders prior to or parallel with the introduction of the malaria vaccine.<br>Considering and addressing of the socio-cultural aspects (religion, ethnicity, occupation and region) that could impede the utilization of the vaccine.	-	Good
Ojakaa DI (2011)	South Coast and Busia	Urban and Rural	Qualitative	Parents, caregivers of children, teachers, the media, Community leaders, local administrators, local government officials, Health care personnel and administrators	Lack of information about the vaccine	Communication strategy to clarify the questions and expectations of stakeholders prior to or parallel with the introduction of the malaria vaccine.<br>Considering and addressing of the socio-cultural aspects (religion, ethnicity, occupation and region) that	Criterion-based sampling techniques to meet objectives, generalizability of results, social desirability bias.	Fair
Ojakaa DI (2014)	8 Provinces	Urban and Rural	Cross-sectional	Caregiver of children	Level of education, regions with generally low vaccine acceptance,	Target specific segments of child caregivers with relevant messages (residents of regions with low acceptance, service providers in health facilities, older caregivers, less educated)	-	Fair
