# Supplementary material for: Current challenges and proposed solutions to the effective implementation of the RTS, S/AS01 Malaria Vaccine Program in sub-Saharan Africa: A systematic review
Source: PLoS One. 2018 Dec 31;13(12):e0209744. doi: 10.1371/journal.pone.0209744 (PMC6312235; doi:10.1371/journal.pone.0209744)
Supplement: S2 Table — (PDF) [file pone.0209744.s003.pdf]

S2 Table. Qualitative Study Quality Assessment using the critical appraisal skills program (CASP) checklist

Criteria	Angwenyi (2014)	Bingham (2012)	Febir (2013)	Meñaca (2014)	Mtenga (2016)	Ojaka (2011)
1. Was there a clear statement of the aims of the research?	Yes	Yes	Yes	Yes	Yes	Yes
2. Is a qualitative methodology appropriate?	Yes	Yes	Yes	Yes	Yes	Yes
3. Was the research design appropriate to address the aims of the research?	Yes	Yes	Yes	Yes	Yes	Yes
4. Was the recruitment strategy appropriate to the aims of the research?	Yes	Yes	Yes	Yes	Yes	Yes
5. Was the data collected in a way that addressed the research issue?	Yes	Yes	Yes	Yes	Yes	Yes
6. Has the relationship between researcher and participants been adequately considered?	Can’t tell	No	No	Yes	Can’t tell	No
7. Have ethical issues been taken into consideration?	Yes	Yes	Yes	Yes	Yes	Yes
8. Was the data analysis sufficiently rigorous?	Yes	Can’t tell	No	Yes	Yes	Can’t tell
9. Is there a clear statement of findings?	Yes	Yes	Yes	Yes	Yes	Yes
10. How valuable is the research?	Very	Very	Very	Very	Very	Very
Overall risk of bias	Minimal	Minimal	Moderate	Minimal	Minimal	Moderate
Overall Rating/Comment	**Good**	**Good**	**Fair**	**Good**	**Good**	**Fair**
