# Supplementary material for: Current challenges and proposed solutions to the effective implementation of the RTS, S/AS01 Malaria Vaccine Program in sub-Saharan Africa: A systematic review
Source: PLoS One. 2018 Dec 31;13(12):e0209744. doi: 10.1371/journal.pone.0209744 (PMC6312235; doi:10.1371/journal.pone.0209744)
Supplement: S3 Table — (PDF) [file pone.0209744.s004.pdf]

S3 Table. Quality Assessment Tool for the Randomized Trial

Criteria	Angwenyi (2014)
1. Was the study described as randomized, a randomized trial, a randomized clinical trial, or an RCT?	Yes
2. Was the method of randomization adequate (i.e., use of randomly generated assignment)?	Yes
3. Was the treatment allocation concealed (so that assignments could not be predicted)?	Yes
4. Were study participants and providers blinded to treatment group assignment?	Yes
5. Were the people assessing the outcomes blinded to the participants' group assignments?	Yes
6. Were the groups similar at baseline on important characteristics that could affect outcomes (e.g., demographics, risk factors, co-morbid conditions)?	Yes
7. Was the overall drop-out rate from the study at endpoint 20% or lower of the number allocated to treatment?	No
8. Was the differential drop-out rate (between treatment groups) at endpoint 15 percentage points or lower?	Yes
9. Was there high adherence to the intervention protocols for each treatment group?	Yes
10. Were other interventions avoided or similar in the groups (e.g., similar background treatments)?	Yes
11. Were outcomes assessed using valid and reliable measures, implemented consistently across all study participants?	Yes
12. Did the authors report that the sample size was sufficiently large to be able to detect a difference in the main outcome between groups with at least 80% power?	Yes
13. Were outcomes reported or subgroups analyzed prespecified (i.e., identified before analyses were conducted)?	Yes
14. Were all randomized participants analyzed in the group to which they were originally assigned, i.e., did they use an intention-to-treat analysis?	Yes
Overall Rating	**Good**
